# Draft Genome Sequence of a Copper-Resistant Marine Bacterium, *Pantoea agglomerans* Strain LMAE-2, a Bacterial Strain with Potential Use in Bioremediation

**DOI:** 10.1128/genomeA.00525-16

**Published:** 2016-06-16

**Authors:** Gino Corsini, Natalia Valdés, Paulina Pradel, Mario Tello, Luis Cottet, Laura Muiño, Eduardo Karahanian, Antonio Castillo, Alex R. Gonzalez

**Affiliations:** a Centro de Investigación Biomédica, Facultad de Ciencias de la Salud, Instituto de Ciencias Biomédicas, Universidad Autónoma de Chile, Santiago, Chile; b Facultad de Química y Biología, Unidad de Apoyo Bioinformatico, Universidad de Santiago de Chile, Santiago, Chile; c Laboratorio de Fisiología y Biologia Molecular Vegetal, Facultad de Ciencias Agropecuarias y Forestales, Universidad de la Frontera, Temuco, Chile; d Centro de Biotecnología Acuícola, Departamento de Biología Facultad de Química y Biología, Universidad de Santiago de Chile, Santiago, Chile; e Departamento de Biología, Facultad de Química y Biología, Laboratorio de Virología de Hongos, Universidad de Santiago de Chile, Santiago, Chile; f Departamento de Ciencias Biológicas y Biodiversidad, Laboratorio de Microbiología Ambiental y Extremófilos, Universidad de Los Lagos, Los Lagos, Chile; g Universidad Científica del Sur, Lima, Perú

## Abstract

*Pantoea agglomerans* LMAE-2 was isolated from seabed sediment moderately contaminated with Cu^2+^. Here, we report its draft genome sequence, which has a size of 4.98 Mb. The presence of *cop* genes related with copper homeostasis in its genome may explain the resistance and strengthen its potential for use as bioremediation agent.

## GENOME ANNOUNCEMENT

*Pantoea agglomerans* is a Gram-negative, motile, bacillus-shaped, aerobic bacterium commonly isolated from several environments, such as plants, soil, and water (1–3). The *Pantoea agglomerans* strain LMAE-2 was isolated from marine sediment moderately contaminated with copper in Tenglo Channel, Puerto Montt, Chile. This strain was able to grow in culture medium containing 9 mM Cu^2+^, with this copper concentration being higher than other species of the *Pantoea* genus can withstand (4). The bacterial resistance to Cu^2+^ is highly important, as a few research groups have been focused on quantifying the copper resistance levels by members of the *Pantoea* genus (2–6).

Copper is an essential microelement for life but at high concentrations can produce cellular damage and detrimental effects on the environment. Studies have been conducted in *P. agglomerans* LMAE-2 to try to understand its exceptional resistance and sorption to copper, both phenomena unusual in this species of marine neutrophil bacterium (4).

The genomic DNA of strain LMAE-2 was sequenced using the Illumina MiSeq system technology based on sequencing by synthesis (SBS). This sequencing platform generates 2 × 250-bp reads (paired-end), obtaining 2 × 5,254,345 reads. Read analysis was performed by FastQC version 0.10.1 (http://www.bioinformatics.bbsrc.ac.uk/projects/fastqc/), and low-quality sequences were removed before assembly using Trimmomatic version 0.32. Trimmed sequences were assembled *de novo* using assembled coverage of 155×, with Velvet assembler version 1.2.10 software. Annotation was performed using RAST, identifying 4,669 genes encoding proteins. In addition, 96 genes encoding RNA were identified. tRNA identification was performed by tRNAscan-SE and rRNA identification by RNAmmer 1.2 server. Proteins identified by RAST were used to determine the Clusters of Orthologous Genes (COG) through the WebMGA server (7), clustered regularly interspaced short palindromic repeats (CRISPRs) were evaluated by CRISPRFinder (8), transmembrane helix domains were determined by TMHMM 2.0c, and signal peptides were estimated by SignalP 4.0 (9). Assembly of the *P. agglomerans* LMAE-2 genome resulted in 155 contigs, with sizes ranging from 233 to 467,377 bp (*N*_50_, 152,429 bp). The total draft genome of *P. agglomerans* LMAE-2 has 4,981,165 bp, with a G+C content of 55.25%. Furthermore, it contains 80 tRNA sequences, three complete rRNA operons (5S-16S-23S), and 17.37% of genes with unknown function.

The results of the genome sequencing revealed that the LMAE-2 strain has a genes cluster, *copABCD*, similar to operons found in other bacterial species and whose gene products are involved in copper resistance (10, 11). The presence of these genetic components in the LMAE-2 strain could be pathway to reach the high resistance level and their capacity for metal sorption, which were previously detected (4). The biological capacity observed in this bacterium offers future perspectives focused on environmental remediation.

### Nucleotide sequence accession numbers.

The draft genome sequence of the LMAE-2 strain has been deposited in the DDBJ/ENA/GenBank under the accession no. JWLQ00000000. The bacterial strain was deposited in Chilean Collection of Genetic Microbial Resources (http://www.cchrgm.cl) under access code RGM 2222.
